# The Tumor Microenvironment Modulates Choline and Lipid Metabolism

**DOI:** 10.3389/fonc.2016.00262

**Published:** 2016-12-22

**Authors:** Noriko Mori, Flonné Wildes, Tomoyo Takagi, Kristine Glunde, Zaver M. Bhujwalla

**Affiliations:** ^1^JHU ICMIC Program, Division of Cancer Imaging Research, The Russell H. Morgan Department of Radiology and Radiological Science, Baltimore, MD, USA; ^2^Sidney Kimmel Comprehensive Cancer Center, School of Medicine, The Johns Hopkins University, Baltimore, MD, USA

**Keywords:** choline metabolism, lipid metabolism, choline kinase, breast cancer, prostate cancer, cell culture, xenograft model

## Abstract

An increase of cellular phosphocholine (PC) and total choline (tCho)-containing compounds as well as alterations in lipids have been consistently observed in cancer cells and tissue. These metabolic changes are closely related to malignant transformation, invasion, and metastasis. The study of cancer cells in culture plays an important role in understanding mechanisms leading to altered choline (Cho) and lipid metabolism in cancer, as it provides a carefully controlled environment. However, a solid tumor is a complex system with a unique tumor microenvironment frequently containing hypoxic and acidic regions and areas of nutrient deprivation and necrosis. Cancer cell–stromal cell interactions and the extracellular matrix may also alter Cho and lipid metabolism. Human tumor xenograft models in mice are useful to mimic the growth of human cancers and provide insights into the influence of *in vivo* conditions on metabolism. Here, we have compared metabolites, obtained with high resolution ^1^H MRS of extracts from human breast and prostate cancer cells in a 2-dimensional (2D) monolayer culture and from solid tumor xenografts derived from these cells, as well as the protein expression of enzymes that regulate Cho and lipid metabolism. Our data demonstrate significant differences in Cho and lipid metabolism and protein expression patterns between human breast and prostate cancer cells in culture and in tumors derived from these cells. These data highlight the influence of the tumor microenvironment on Cho and lipid metabolism.

## Introduction

A solid tumor is a complex system with a unique microenvironment that frequently contains areas of hypoxia, extracellular acidosis, and necrosis (1). Cancer cell–stromal/endothelial cell interactions and nutrient deprivation are some of the additional factors that influence metabolism in solid tumors (1–3). Although investigating cancer cell metabolism using cells in culture has the advantages of rapid use and lower costs, it is important to validate these results with tumor studies because of the complexities of solid tumor microenvironments that may alter metabolism and gene expression profiles compared to cells in culture. This is important in the development of biomarkers and in identifying targets for cancer treatment.

Cancer cells display aberrant choline (Cho) and lipid metabolism. Phosphatidylcholine (PtdCho), the most abundant phospholipid in eukaryotic cell membranes, contributes to proliferative growth and programed cell death (4). High levels of cellular phosphocholine (PC) and total choline-containing compounds [tCho: the sum of Cho, PC, and glycerophosphocholine (GPC)] have been consistently observed in cancer cells and tumor tissue and are closely related to malignant transformation, invasion, and metastasis (5–12).

Among the enzymes that regulate Cho metabolism, overexpression of choline kinase (Chk), the enzyme that catalyzes the phosphorylation of Cho to yield PC in the first step of PtdCho biosynthesis (Kennedy pathway) (13, 14), is a major cause of increased PC and tCho observed in cancers (8, 11, 15). The elevated tCho level detected by ^1^H magnetic resonance spectroscopy (MRS) is being evaluated as a specific biomarker of prostate cancer, and high tCho is associated with aggressiveness in breast cancer (11, 16). Downregulation of Chk-α has been shown to significantly reduce proliferation in breast cancer cells (17, 18) and tumors (9). Enzymes that control the catabolism of PtdCho include phospholipase A (PLA), PLC, and PLD. These enzymes maintain PtdCho levels. Cytosolic phospholipase A_2_ (cPLA_2_α) has significantly different expression levels in basal-like and luminal-like breast cancer xenografts (19). PLD_1_ is upregulated in various human cancers, including breast (20, 21), uterine (22), and endometrial (23) cancers. An association between PLD_1_ and Chk-α expression with breast cancer malignancy was recently observed (21). Deregulated Cho phospholipid metabolism is emerging as a metabolic hallmark of oncogenesis and tumor progression.

Lipids function as energy storage molecules, structural components of cell membranes, and signaling molecules involved in cell growth, inflammation, and immunity (24, 25). Increased lipid biosynthesis is a characteristic feature of cancer. Elevated *de novo* fatty acid synthesis is necessary for rapidly proliferating tumor cells to continually provide lipids, such as phospholipids, for membrane production. Proton spectroscopy of lipid-soluble cancer cell and tumor extracts detects signals from fatty acids, cholesterol, and phospholipids. Fatty acid synthase (FASN) is an important lipogenic enzyme required for fatty acid synthesis. FASN overexpression has been reported in several human cancers including breast, prostate, colon, and ovary and has been associated with poor prognosis (26–32).

Here, we obtained high resolution ^1^H MR spectra of extracts from cancer cells, and the corresponding tumor xenografts derived from these cells, to identify differences in Cho and lipid metabolism between cells and tumors. We selected two human prostate (DU-145 and PC-3) and two human breast (MCF-7 and MDA-MB-231) cancer cell lines with different aggressiveness for these studies. Expression of Chk-α, cPLA_2_, PLD_1_, and FASN was characterized in cells and tumors to understand the molecular mechanisms underlying the differences in Cho and lipid metabolism observed between cells and tumors. Significant differences in Cho metabolites, especially PC and tCho, were observed between cells and tumors that were reflected in the differences in enzyme expression. These results underline the importance of the tumor microenvironment and conditions that exist *in vivo*, in modulating the Cho and lipid metabolism of cells in tumors, and provide new insights into the regulation of these metabolic pathways.

## Materials and Methods

### Cell Culture

Two prostate and two breast cancer cell lines were used in this study. PC-3 and DU-145 prostate cancer cells are both androgen independent, but PC-3 is more invasive and metastatic than DU-145 prostate cancer cells (33). MDA-MB-231 is a triple negative metastatic human breast cancer cell line, and MCF-7 is an estrogen receptor/progesterone receptor-positive poorly metastatic human breast cancer cell line (33). All cell lines were obtained from American Type Culture Collection (Manassas, VA, USA) and were maintained in a humidified atmosphere with 5% CO_2_ in air, at 37°C. The cell lines were grown in RPMI-1640 medium supplemented with 10% fetal bovine serum (Sigma-Aldrich, St. Louis, MO, USA) and 100 units/ml penicillin and 100 µg/ml streptomycin (Life Technologies Ltd., Grand Island, NY, USA).

### Generation of Tumor Xenografts

Approximately 2 × 10^6^ cells in 50 µL Hanks’ balanced salt solution (Sigma-Aldrich, St. Louis, MO, USA) were inoculated in the mammary fat pad (breast cancer cells) or the flank (prostate cancer cells) of severe combined immunodeficient mice. For MCF-7 tumors, a 17 β-estradiol pellet (0.18 mg 90-day release pellet, Innovative Research of America, Sarasota, FL, USA) was implanted subcutaneously 2 days prior to cancer cell inoculation. Mice were fed with Teklad global 18% protein extruded rodent diet (Harlan, Madison, WI, USA) that includes 1,200 mg/kg of Cho, 0.9% total saturated fatty acids, and 4.7% of total unsaturated fatty acids with minerals, amino acids, vitamins, and no cholesterol.

### Dual-Phase Extraction of Cells and Tumors

Cells were cultured to about 80% confluence, and medium was changed 3 h prior to cell collection to avoid any lack of nutrition. Adherent cells were collected by trypsinization and counted using a hemocytometer after staining dead cells with trypan blue. Approximately 4–5 × 10^7^ cells were harvested for cell extraction. Solid tumors were excised at volumes of ~200–500 mm^3^ (~0.2–0.4 g) and immediately freeze-clamped in liquid N_2_. The time from cell inoculation to tumor excision was approximately 50 days for DU-145, 30 days for PC-3, 40 days for MCF-7, and 60 days for MDA-MB-231 tumors.

Both lipid- and water-soluble extract fractions were obtained using a dual-phase extraction method as described previously (17). Briefly, pelleted cells were mixed with 4 mL of ice-cold methanol and vigorously vortexed. For tumor samples, ground tumors in liquid N_2_ were mixed with 4 mL of ice-cold methanol and homogenized. After keeping samples on ice for 15 min, 4 mL of chloroform were added, vortexed vigorously, and kept on ice for 10 min. Finally, 4 mL of water were added and shaken well. All operations were performed on ice, and samples were stored at 4°C overnight for phase separation and later centrifuged at 15,000 *g* at 4°C for 30 min. The water/methanol phase containing water-soluble cellular metabolites such as Cho, PC, and GPC were treated with ~100 mg of chelex beads (Sigma-Aldrich, St. Louis, MO, USA) to remove any divalent cations. After removing the beads, methanol was evaporated using a rotary evaporator. The remaining water phase was lyophilized. The chloroform phase (lipid-soluble phase) was collected in the tube, and chloroform was evaporated using nitrogen gas. Both phases of the extracts were stored at −20°C until use.

### Magnetic Resonance Spectroscopy

Water-soluble extracts from cells and tumors were resuspended in 0.6 mL of deuterated water (D_2_O) containing 2.4 × 10^−7^ mol of 3-(trimethylsilyl)propionic 2,2,3,3-d_4_ acid (TSP; Sigma-Aldrich, St. Louis, MO, USA) as an internal standard for MR spectral analysis. Lipid-soluble extracts were resuspended in 0.4 mL of chloroform-D and 0.2 mL of methanol-D4 with 0.05 v/v% tetramethylsilane (TMS) (Cambridge Isotope Laboratories, Inc., Tewksbury, MA, USA) as an internal standard. Fully relaxed ^1^H MR spectra of water-soluble extracts and lipid-soluble extracts were acquired on a Bruker Avance 11.7 T spectrometer (Bruker BioSpin Corp., Billerica, MA, USA) with flip angle = 30°, sweep width = 10,000 Hz, repetition time = 11.2 s, block size = 32 K, and scans = 128. MR spectra were analyzed using Bruker XWIN-NMR 3.5 software (Bruker BioSpin) as previously described (34). Signal integrals of –N^+^(CH_3_)_3_ resonances of PC at ~3.226 ppm, GPC at ~3.235 ppm, and free Cho at ~3.208 ppm in water-soluble extracts were determined and normalized to cell number and cell volume and compared to the TSP standard. To determine concentrations of cell samples, peak integration (*I*_met_) from ^1^H spectra of PC, GPC, and Cho were compared to that of the internal standard TSP (*I*_TSP_) according to the equation:

(1) $$[{\text{metabolite}}_{\text{cell}}]=A_{\text{TSP}}\cdot \frac{I_{\text{met}}/H}{(I_{\text{TSP}}/H)\cdot N_{\text{cell}}\cdot V_{\text{cell}}}$$

(2) $$[{\text{metabolite}}_{\text{tumor}}]=A_{\text{TSP}}\cdot \frac{I_{\text{met}}/H}{(I_{\text{TSP}}/H)\cdot V_{\text{tumor}}}$$

In these equations, [metabolite_cell_] represents the intracellular concentration of the metabolite of interest expressed as mole per liter (M), *A*_TSP_ is the number of moles of TSP (2.4 × 10^−7^ mol) in the sample, *H* is the number of protons contributing to the signal, *N*_cell_ is the cell number, and *V*_cell_ is the cell volume. To determine the cell volume, cell size was determined by trypsinizing the cells and measuring the diameter (d) of 100 randomly selected cells using an optical microscope and calculated as [(4π/3) × (d/2)^3^]. The cell volumes used for calculation are MDA-MB-231: 2,050 µm^3^, MCF-7: 3,128 µm^3^, PC-3: 3,120 µm^3^, and DU-145: 3,630 µm^3^ (7).

Tumor metabolite concentration [metabolite_tumor_] was calculated in mole per liter (M), *V*_tumor_ is the tumor volume in liter (assuming 1 g = 1 mL).

In lipid phase samples, chemical shifts were referenced to the internal standard TMS resonance at 0 ppm using published data (35–38). To determine the arbitrary unit (A.U.) of metabolites from lipid phase samples, signal integrals (*I*_met_) of methyl groups assigned to C_18_ of cholesterol (C_18_) at ~0.7 ppm, methyl groups (–CH_3_) at ~0.9 ppm, methylene groups (–(CH_2_)n–) at ~1.3 ppm, and ethylene groups (–CH = CH–) at ~5.4 ppm in acyl chains of lipids, –CH_2_–N in phosphatidylethanolamine (PtdE) at ~3.1 ppm, the Cho group (–N^+^(CH_3_)_3_) primarily from PtdCho at ~3.2 ppm peaks were determined and compared to that of TMS (*I*_TMS_). A.U. values were standardized by cell number (*N*_cell_) or tumor weight (g) (*W*_tumor_). For the comparison of cells and tumors, ratios of A.U. values were used.

(3) $$\text{A.U./cell}=\frac{I_{\text{met}}\cdot 10^{9}}{I_{\text{TMS}}\cdot N_{\text{cell}}}$$

(4) $$\text{A.U./g}=\frac{I_{\text{met}}\cdot 10}{I_{\text{TMS}}\cdot W_{\text{tumor}}}$$

### Immunoblot Analysis

Cells were grown in culture medium and scraped into RIPA buffer [50 mM Tris, pH 7.4, 150 mM NaCl, 1 mM EDTA, 1% Triton-100, 1% sodium deoxycholate, 1 mM phenylmethylsulfonyl fluoride, 0.1% SDS, and a protease inhibitor cocktail at 1:200 dilution (Sigma-Aldrich, St. Louis, MO, USA)], and cell lysates were incubated on ice for 30 min. Protein samples from tumors were prepared after grinding freeze-clamped tumors in lipid N_2_ and sonicating in Hepes buffer with a protease inhibitor cocktail. Cell and tumor lysates were spun down at 16,000 *g* (refrigerated centrifuge 5415 R, Eppendorf, Westbury, NY, USA) and 4°C twice. Protein concentrations were estimated using the Bio-Rad DC assay (Bio-Rad, Hercules, CA, USA). Equal amounts of total protein (40 or 50 µg) from cells or tumors were resolved on one-dimensional 7.5% SDS-PAGE gels and transferred to a nitrocellulose membrane (Bio-Rad). After blocking in 5% milk-TBST (TBS Tween), the membrane was separately probed with a custom-made polyclonal Chk-α antibody (Proteintech Group, Inc., Chicago, IL, USA) (17), cPLA_2_ antibody (Santa Cruz Biotechnology, Inc. Dallas, TX, USA), FASN antibody (Santa Cruz Biotechnology), and PLD_1_ antibody (Abcam, Cambridge, MA, USA). Anti-GAPDH antibody (Molecular Probes, Eugene, OR, USA) was used for equal loading assessment. Secondary antibodies were horseradish peroxidase conjugated anti-mouse or anti-rabbit IgG (Vector Laboratories, Burlingame, CA, USA). Reactions were recorded on Blue Bio film (Denville Scientific, Metuchen, NJ, USA) following use of Super Signal West Pico Substrate (Pierce Biotech, Rockford, IL, USA).

### Statistical Analysis

Data were expressed as mean ± SD. The statistical significance was evaluated using a two-tailed unpaired Student’s *t*-test. *P* values of less than 0.05 were considered to be significant unless otherwise stated. Four or more samples were used for cell culture data and tumor data.

## Results

### Levels of Cho Metabolites in Water-Soluble Extracts Determined by MRS

**Figure 1:**
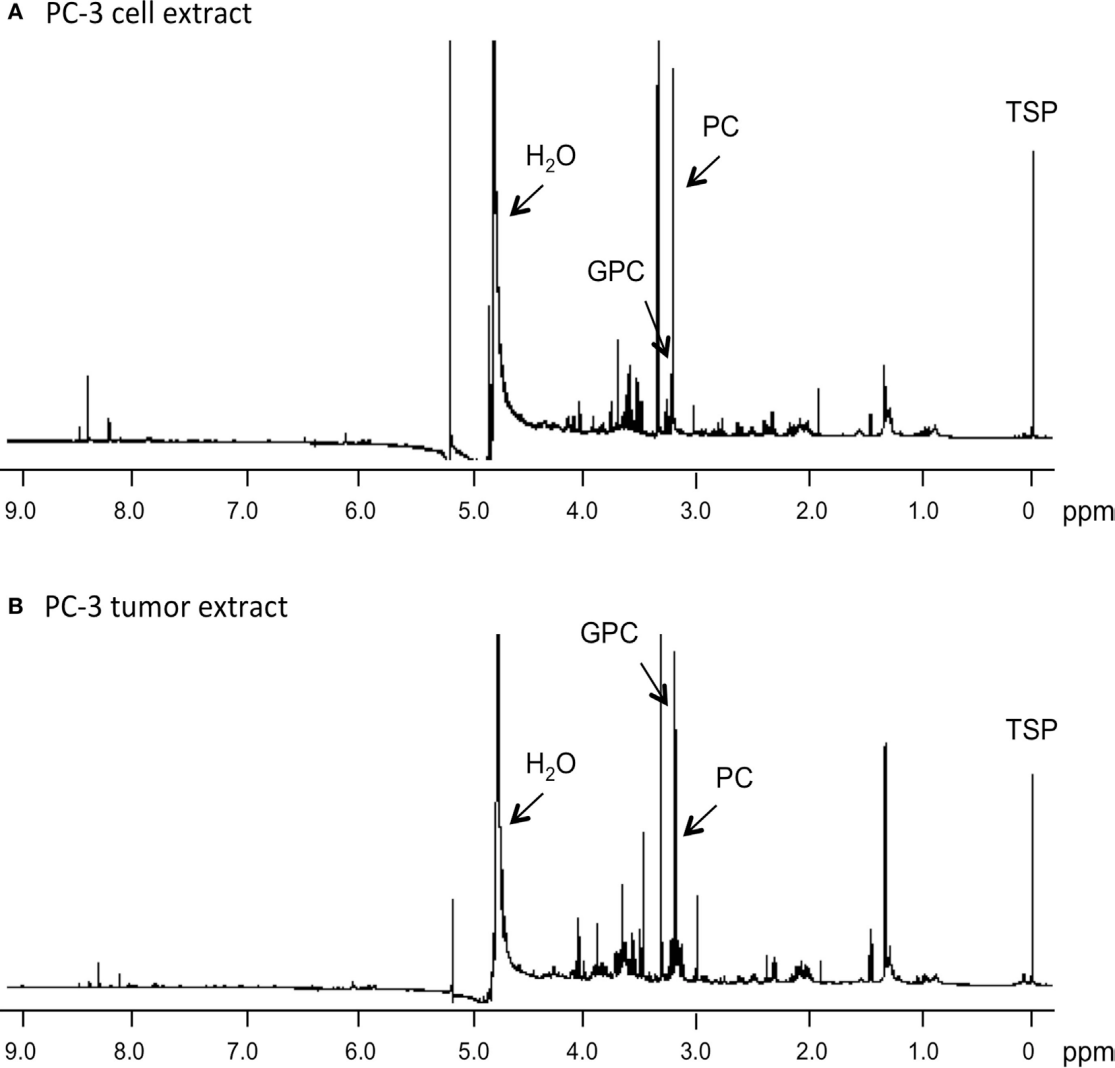
**Representative ^1^H MR spectra obtained from water-soluble extracts of (A) PC-3 cells (4 × 10^7^ cells) and (B) PC-3 tumor xenograft (0.4 g)**. Spectra were acquired on a Bruker Avance 11.7 T spectrometer with a 30° flip angle, 10,000 Hz sweep width, 11.2 s repetition time, 32 K block size, and 128 scans. TSP, 3-(trimethylsilyl)propionic 2,2,3,3-d_4_ acid, an internal standard at 0 ppm; PC, phosphocholine; GPC, glycerophosphocholine; H_2_O, water signal.

**Figure 2:**
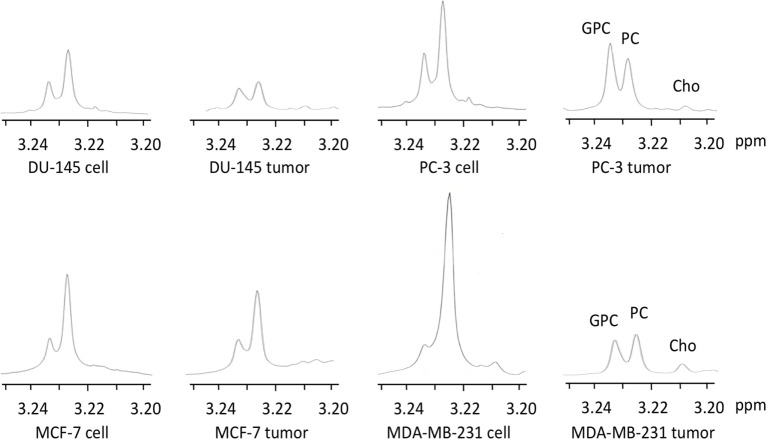
**Representative choline metabolite regions of ^1^H MR spectra obtained from water-soluble extracts of DU-145, PC-3, MCF-7, and MDA-MB-231 cells and the corresponding tumor xenografts**. Spectra are expanded to display signals from 3.20 to 3.25 ppm. Peak assignments are: free Cho at ~3.208 ppm, PC at ~3.226 ppm, and GPC at ~3.235 ppm.

**Figure 3:**
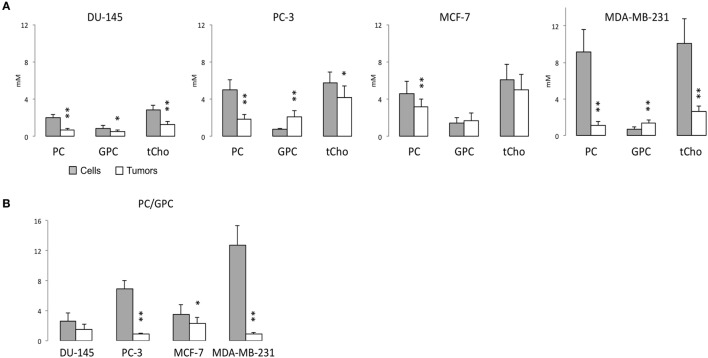
**(A)** PC, GPC, and total choline (tCho) (PC + GPC + Cho) levels, and **(B)** PC/GPC in water-soluble extracts obtained from ^1^H MR spectra of DU-145, PC-3, MCF-7, and MDA-MB-231 cells (gray bar) or the corresponding tumor xenografts (white bar). Average cell number used for water-soluble extracts are DU-145: 4.09 ± 1.00 × 10^7^ (*n* = 7), PC-3: 3.85 ± 0.40 × 10^7^ (*n* = 6), MCF-7: 4.84 ± 0.99 × 10^7^ (*n* = 10), and MDA-MB-231: 4.55 ± 0.93 × 10^7^ (*n* = 4). Average tumor weights in gram used for water-soluble extracts are DU-145: 0.31 ± 0.07 (*n* = 8), PC-3: 0.34 ± 0.08 (*n* = 7), MCF-7: 0.28 ± 0.09 (*n* = 11), and MDA-MB-231: 0.31 ± 0.05 (*n* = 7). Data represent mean ± SD. **P < 0.01, *P < 0.05 between cells and tumors.

Representative ^1^H MR spectra of water-soluble metabolites obtained from PC-3 cells and a tumor extract are shown in Figure 1. Expanded Cho metabolite regions of water-soluble cell and tumor extract spectra from DU-145, PC-3, MCF-7, and MDA-MB-231 cell lines are shown in Figure 2 to demonstrate the differences in the pattern of Cho metabolites between the cell and tumor pairs. Data averaged over four cell studies and six tumors showed a consistent decrease of PC in tumors compared to cells in all four cell lines (Figure 3A). The two most aggressive cell lines (PC-3 and MDA-MB-231) showed the strongest decline of PC in tumors compared to cells. GPC tended to increase in tumors compared to cells in PC-3 and MDA-MB-231 with the exception of DU-145 cells where there was a significant decrease in tumors. The several fold decrease of PC overshadowed the small increase of GPC in tumors compared to cells, resulting in a significant decrease of tCho in PC-3, and MDA-MB-231 tumors compared to the cells. In DU-145 tumors, the decrease of both PC and GPC resulted in a significant decrease of tCho in tumors compared to the cells.

The PC/GPC ratio was higher in cells than in tumors (Figure 3B), and the differences were larger in the more malignant cell lines (PC-3 and MDA-MB-231) compared to the less malignant cell lines (DU-145 and MCF-7). Interestingly, the higher PC/GPC ratio observed in the more aggressive cell lines (PC-3 and MDA-MB-231) compared to the less aggressive cell lines (DU-145 and MCF-7) was not observed in tumor extracts.

### Levels of Lipid Metabolites in Lipid-Soluble Extracts Determined by MRS

**Figure 4:**
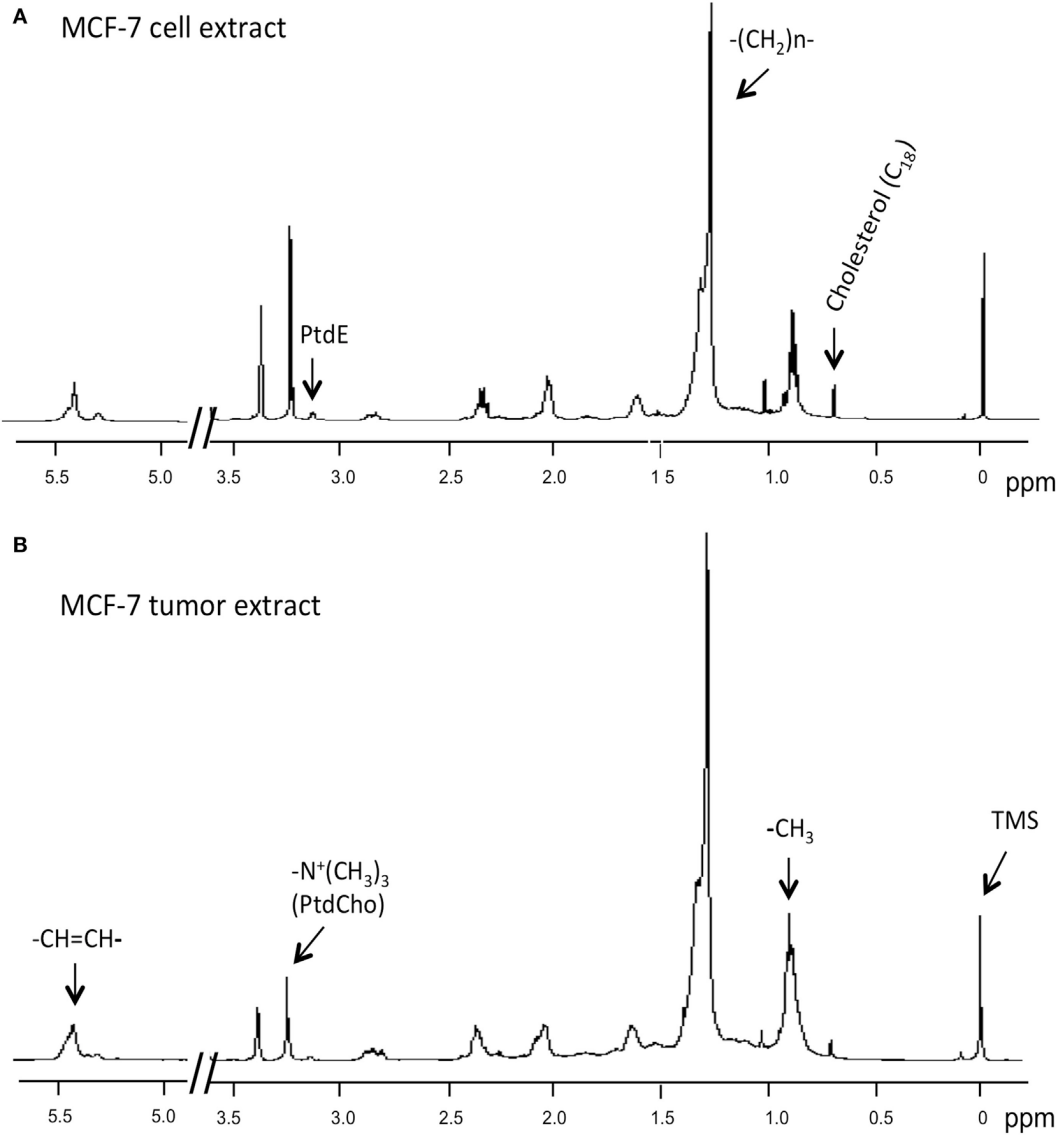
**Representative ^1^H MR spectra obtained from lipid-soluble extracts of (A) MCF-7 cells and (B) MCF-7 tumor xenograft**. Spectra were acquired on a Bruker Avance 11.7 T spectrometer and are expanded to display signals from 0 to 5.5 ppm (except for 3.7–4.8 ppm). Lipid spectra were acquired with a 30° flip angle, 10,000 Hz sweep width, 11.2 s repetition time, 32 K block size, and 128 scans. Peak assignments are: TMS, tetramethylsilane (internal standard) at 0 ppm, methyl groups assigned to C_18_ of cholesterol (C_18_) at ~0.7 ppm, the methyl groups (–CH_3_) at ~0.9 ppm, methylene groups (–(CH_2_)n–) at ~1.3 ppm, and ethylene groups (–CH=CH–) at ~5.4 ppm in acyl chains of lipids, –CH_2_–N in phosphatidylethanolamine at ~3.1 ppm, and the choline group (–N^+^(CH_3_)_3_) primarily from phosphatidylcholine at ~3.2 ppm.

**Figure 5:**
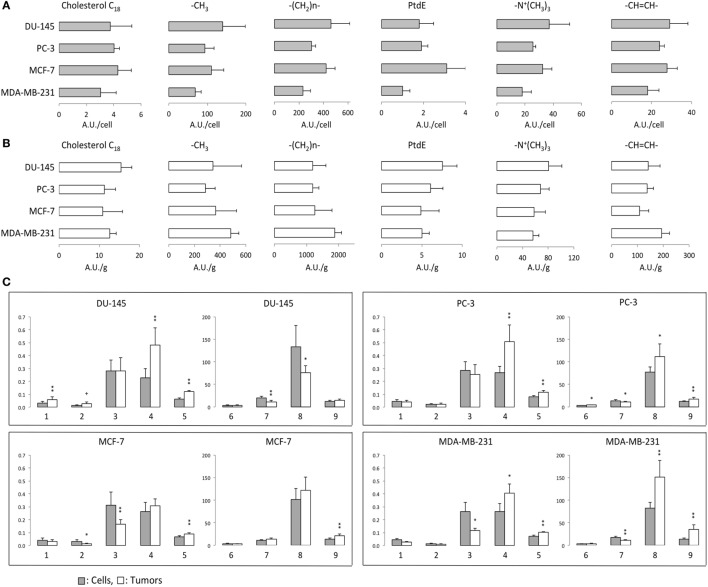
**(A)** Lipid metabolites in arbitrary unit (A.U.)/cell from ^1^H MR spectra obtained from lipid-soluble extracts of DU-145, PC-3, MCF-7, and MDA-MB-231 cells. **(B)** Lipid metabolites in A.U./g from ^1^H MR spectra obtained from lipid-soluble extracts of DU-145, PC-3, MCF-7, and MDA-MB-231 xenograft models. **(C)** Ratios of lipid resonances from cells (gray bar) and solid tumors (white bar). 1: C_18_/–CH_3_, 2: phosphatidylethanolamine (PtdE)/–CH_3_, 3: –N^+^(CH_3_)_3_/–CH_3_, 4: –CH=CH–/–CH_3_, 5: –CH=CH–/–(CH_2_)n–, 6: –(CH_2_)n–/–CH_3_, 7: –N^+^(CH_3_)_3_/PtdE, 8: –(CH_2_)n–/C_18_, 9: –(CH_2_)n–/–N^+^(CH_3_)_3_. Average cell number used for lipid extracts are DU-145: 4.30 ± 0.96 × 10^7^ (*n* = 6), PC-3: 3.85 ± 0.40 × 10^7^ (*n* = 6), MCF-7: 5.07 ± 0.92 × 10^7^ (*n* = 7), and MDA-MB-231: 4.55 ± 0.93 × 10^7^ (*n* = 4). Average tumor weights in gram used for lipid extracts are DU-145: 0.33 ± 0.07 (*n* = 6), PC-3: 0.34 ± 0.08 (*n* = 7), MCF-7: 0.27 ± 0.09 (*n* = 9), and MDA-MB-231: 0.30 ± 0.05 (*n* = 6). Data represent mean ± SD. **P < 0.01, *P < 0.05, ^+^*P* < 0.06 between cells and tumors.

^1^H MRS was used to compare lipid-soluble metabolites from cells and tumors. Representative ^1^H MR spectra from MCF-7 cells are shown in Figure 4. Data summarized over multiple cell and tumor samples are presented as A.U./cell from cells in culture (Figure 5A) and A.U./g from tumors (Figure 5B). Data from the methyl groups assigned to C_18_ of cholesterol (C_18_), the methyl groups (–CH_3_), the methylene groups (–(CH_2_)n–), and ethylene groups (–CH=CH–) in acyl chains of lipids, –CH_2_–N in PtdE, the Cho group (–N^+^(CH_3_)_3_) primarily from PtdCho are presented. To compare cells and tumors within the same cell lines, metabolite integrals were normalized to the –CH_3_ signal to obtain ratios of C_18_/–CH_3_, –(CH_2_)n–/–CH_3_, PtdE/–CH_3_, –N^+^(CH_3_)_3_/–CH_3_, –CH=CH–/–CH_3_. Additional analysis was performed to obtain ratios of –CH=CH–/–(CH_2_)n–, –N^+^(CH_3_)_3_/PtdE, –(CH_2_)n–/C_18_, and –(CH_2_)n–/–N^+^(CH_3_)_3_ (Figure 5C).

The degree of lipid unsaturation estimated from –CH=CH–/–CH_3_ or –CH=CH–/–(CH_2_)n– was significantly and consistently higher in tumors than in cells, in all the cell lines (Figure 5C). The ratio of –(CH_2_)n–/–CH_3_, which is related to the length of fatty acids, was significantly higher in PC-3 tumors than in cells. Ratios of PtdCho (–N^+^(CH_3_)_3_) to –CH_3_ were higher in cells than in tumors in the breast cancer cell lines. The –N^+^(CH_3_)_3_/PtdE ratios were higher in cells than in tumors in the prostate cancer cell lines and the MDA-MB-231 breast cancer cell line but not in MCF-7 cells. Ratios of –(CH_2_)n–/C_18_ and –(CH_2_)n–/–N^+^(CH_3_)_3_ were higher in tumors than in cells from more malignant PC-3 and MDA-MB-231 cell lines. C_18_/–CH_3_ and –(CH_2_)n–/C_18_ results showed DU-145 tumors had higher ratio of cholesterol in lipids. Both aggressive PC-3 and MDA-MB-231 cell lines showed similar lipid metabolite changes in cells and tumors.

### Levels of Protein That Are Related to Cho Phospholipid Metabolism and Lipid Metabolism

**Figure 6:**
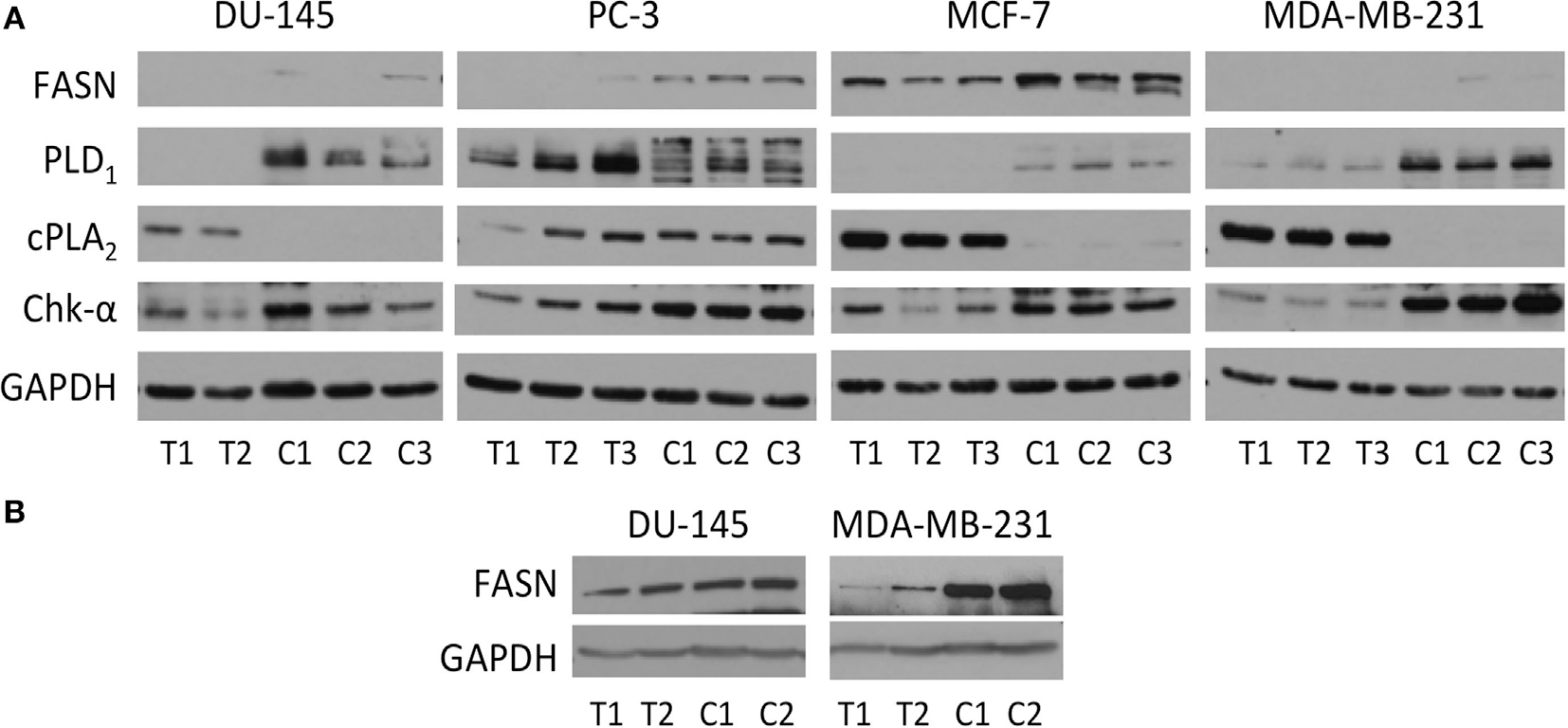
**(A)** Protein levels of Chk-α, cPLA_2_, PLD_1_, and fatty acid synthase (FASN) determined by immunoblot assays of DU-145, PC-3, MCF-7, and MDA-MB-231 cells (marked C) and the tumor xenografts (marked T). **(B)** Protein levels of FASN in DU-145 and MDA-MB-231 cells (C) and the tumor xenografts (T) were obtained from a separate immunoblot assay with different exposure time. 50 µg **(A)** or 40 µg **(B)** of protein was loaded on a 10% reducing SDS-PAGE gel. GAPDH protein levels were used for equal loading assessment.

Immunoblot assays of Chk-α, cPLA_2_, PLD_1_, and FASN antibodies are shown in Figure 6A. A consistently higher expression of Chk-α was observed in cells compared to solid tumors that was most pronounced in MDA-MB-231 cells.

Higher expression of cPLA_2_ was observed in DU-145, MDA-MB-231, and MCF-7 tumors compared to the corresponding cells. Like Chk-α, PLD_1_ was higher in cells than in corresponding tumors with the exception of PC-3 cells.

Overall, levels of FASN were higher in cells than in tumors. The levels of FASN were higher in PC-3 and MCF-7 cells than in the corresponding tumors (Figure 6A). Since levels of FASN in DU-145 and MDA-MB-231 were lower than in the other prostate or breast cancer cell lines, these were not detectable on the same gel (Figure 6A). Therefore, samples from DU-145 and MDA-MB-231 cells and tumors were loaded separately and run with different exposure time (Figure 6B). MDA-MB-231 had higher level of FASN protein in cells than in tumors, and DU-145 had slightly higher or comparable level of FASN in cells than in tumors.

## Discussion

Here, for the first time, we have compared Cho and lipid metabolites in cells maintained in 2-dimensional (2D) monolayer cell culture to the corresponding tumor xenografts and have performed molecular characterization of some of the major enzymes that regulate Cho and lipid metabolism in these cells and tumors. We observed significant differences in metabolite and enzyme expression patterns between cells and tumors confirming the importance of the tumor microenvironment in modulating metabolism. A compendium of factors including vascularization that control substrate delivery and hypoxia, acidic extracellular pH (pHe), and areas of cell death can influence the enzymes and the metabolites measured in high resolution spectra of tumor extracts.

One common difference to emerge across all the cells lines was a significant decrease of PC and tCho in tumors compared to cells that was consistent with the decrease of Chk-α observed in the tumors compared to the cells. This uniform difference across cell lines is surprising given the differences in vascularization (33), necrosis, hypoxia, and acidosis that have been observed in these tumor models and implicates the high cellular density in tumors, compared to cells in culture, as one mechanism underlying the difference. Additional mechanisms causing the decrease of PC, tCho, and Chk-α in tumors may be related to cancer cell-stromal cell and cancer cell-extracellular matrix (ECM) interactions that are intrinsic to tumor growth *in vivo*.

The second common difference that emerged from the comparison was a decrease of FASN in tumors compared to cells. Again the uniformity of this observation across the cell lines suggests that the reduction of FASN in tumors was cell density, stromal cell, or ECM related. Given that mechanical stress can create significant changes in cell function (39), it is possible that cancer cells growing within an ECM may demonstrate differences in enzyme expression and consequently metabolism. The uniform decrease of FASN observed in the tumors may also explain the higher unsaturated fatty acids observed in tumors compared to cells, in all the cell lines. Although endogenous and exogenous fatty acids are utilized similarly by MDA-MB-231 and MCF-7 cells in culture (40), mouse diet contains higher unsaturated fat (4.7%) than saturated fat (0.9%), which may also contribute to increased unsaturated fatty acids observed in tumors compared to cells.

Another interesting observation that emerged from these studies was that the high PC/GPC ratio associated with aggressive breast and prostate cancer cells in culture (6, 41) was not replicated in tumors. This was primarily because of the significant decrease of PC in tumors compared to cells as well as an increase of GPC in tumors compared to cells. Acidic pHe that is frequently observed in tumors (42) has been shown to decrease PC and increase GPC in cells (43). Further characterization, in the future, of GPC phosphodiesterase, the enzyme that converts GPC into Cho and glycerol-3-phosphate (44, 45) and lysophospholipase, the enzyme that converts lyso-PtdCho to GPC (15), may provide further insights into the differences in GPC between cells and tumors observed here. Increased cell density has also been observed to increase GPC levels (46). We have previously studied PC-3, DU-145, and MDA-MB-231 cells in a cell perfusion system where multilayered cells grow on 3D beads and have observed similar metabolic profiles as observed in 2D culture, especially in PC-3 (47) and MDA-MB-231 cells (48). Although we cannot rule out differences between 2D and 3D culture contributing to the metabolic differences, the differences observed here are more likely due to the tumor microenvironment including cancer cell-stromal cell and ECM interactions.

With the exception of the PC-3 cell line, PLD_1_ was higher in cells compared to tumors, and cPLA_2_ was higher in tumors compared to cells. In our experience (33), PC-3 tumors are the least vascularized in the four models studied here. The absence of a difference in PLD_1_ and cPLA_2_ between the PC-3 cell line compared to the remaining three cell lines may be related to the poor vascularization in PC-3 tumors and the resultant reduction of paracrine signaling from vascular molecules.

Additional characterization, in future studies, of metabolites and cytokines in the tumor interstitial fluid, necrosis, pH, and oxygenation as well as an expanded characterization of the enzymes in Cho and lipid metabolism in culture and in tumors will provide further insights into the mechanisms underlying the differences between cells and tumors observed here. Investigation of metabolite differences between primary tumors and metastatic growth will also provide important insights. Such insights will provide further understanding of the functioning of tumors that is critical to developing biomarkers and treatment strategies targeting metabolism.

## Ethics Statement

All surgical procedures and animal handling were performed in accordance with protocols approved by the Johns Hopkins University Institutional Animal Care and Use Committee and conformed to the Guide for the Care and Use of Laboratory Animals published by the NIH.

## Author Contributions

NM; design of the work, the acquisition, analysis, and interpretation of data for the work; drafting the work; final approval of the version to be published; agreement to be accountable for all aspects of the work. FW and TT; the acquisition, analysis, and interpretation of data for the work; revising it critically for important intellectual content; final approval of the version to be published; agreement to be accountable for all aspects of the work. KG and ZB; design of the work; revising it critically for important intellectual content; final approval of the version to be published; agreement to be accountable for all aspects of the work.

## Conflict of Interest Statement

The authors declare that the research was conducted in the absence of any commercial or financial relationships that could be construed as a potential conflict of interest.

## Abbreviations

Cho, choline; Chk, choline kinase; GPC, glycerophosphocholine; ER, estrogen receptor; FASN, fatty acid synthase; MRS, magnetic resonance spectroscopy; PC, phosphocholine; PL, phospholipase; PR, progesterone receptor; PtdCho, phosphatidylcholine; PtdE, phosphatidylethanolamine; tCho, total choline; TMS, tetramethylsilane; TSP, 3-(trimethylsilyl)propionic 2,2,3,3-d_4_ acid.
